# Geological Surveys of Ohio, Indiana and Michigan

**Published:** 1838

**Authors:** 


### Art. XI.—Geological Surveys of Ohio, Indiana and Michigan.

*Ohio*.—We have before us the "First Annual Report on the Geological Survey of the State of Ohio." It is made by the principal Geologist Mr. W. W. Mather, and embraces contributions from four of his assistants, Dr. Hildreth, Mr. Briggs, Dr. Kirtland, and Mr. Whittlesey. The whole make a pamphlet of 134 pages. This very important work, will henceforth advance rapidly. The chief Geologist is not only well qualified, but entirely devoted, in feeling and purpose to his duties. A large part of the report, is from the pen of Dr. Hildreth, who, we are sorry to learn, has resigned. It relates chiefly to the Muskingum valley. Mr. Briggs' contribution, embraces the Hocking and lower Scioto valleys, in the examination of which, he was assisted by Mr. J. W. Foster. Dr. Kirtland is charged with the department of organized nature, on which he has made a brief preliminary report. Mr. Whittlesey is engaged in the topographical survey. As yet he has made but few admeasurements, to determine the great problem of the general elevation of the surface of the state, above Lake Erie and the Atlantic Ocean; but he has collated the facts previously ascertained, and formed from them the following table, which we extract with a part of the remarks that precede it, as being of interest, not merely to our readers in Ohio, but in Kentucky, Indiana, and Illinois; states which bear to the Lakes a relation not unlike that of Ohio.

"The surface of Lake Erie, 564 feet above tide-water, at Albany, is made the plane of reference. This comparison of heights exhibits almost the entire surface of our territory above the level of the Lake. The Ohio river, at Cincinnati, in its lowest stage, is but 133 feet below (that is, nearer the Earth's centre, than) the Lake's surface. Ascending the river to Portsmouth, it rises 37 feet, being 96 beneath the Lake; to Marietta 94 feet, within two feet of the line or plane of reference; and at Beaver, Pennsylvania, its elevation above the waters of Erie, is 127 feet, according to the best information. The highest annual floods lessen the difference between the river below Marietta and the Lake, about 50 feet, and increase the same as we ascend. But the increase of difference is not equal to the decrease, as each tributary, when all discharge surplus water at the same time, of course gives additional height to its waters; yet the extraordinary inundation of February, 1832, which H. G. Eastin, Esq. an Engineer in the employ of the State of Kentucky, puts at 61 feet in the vicinity of the mouth of Big Sandy, was only one foot higher at Cincinnati. From Beaver to Cincinnati, by river, 420 miles, the descent is 260 feet, or 619-1000ths of a foot per mile. But the fall is not uniform, being greatest in the upper part of the river, thus: From Beaver to Marietta, it is 0,86 of a foot per mile; Marietta to Portsmouth, 0,563; Portsmouth to Cincinnati, 0,359. The upper level of the Canal, in the northern part of Cincinnati, is only 21 feet lower than the lake; the intersection of Main and Cross streets, Aberdeen (opposite Maysville,) 28; and the sill of the Old Court-House, at Portsmouth, in Market street, (about 100 feet north of Front,) is but 35 feet below the line of reference, made use of on the sheet of projections. The elevations of the bluffs and knobs bordering on the Ohio river, have not been taken this season, for reasons above given. They have an apparant height of 300 and 400 feet above our general standard (the Lake,) leaving only a small portion of the immediate valleys of the Ohio, the Scioto and the Miamies, beneath it. The sill of the Court-House , at Piketon, is above, a few inches less than 8 feet. The "Little Mountain," a singular elevation in Mentor, Geauga county, about 5 miles from Lake Erie, and the highest point in the northeastern part of the State, rises but 5 feet above Somerset, in Perry county, and 40 feet higher than the doorsill of Hillsborough Court-House, in Highland county, in the southeastern section of Ohio. This striking feature of uniformity in the general surface of the State, cannot be more plainly exhibited, than by a tabular statement, drawn from the materials hitherto collected, showing in contrast, some points, remote from each other, but of corresponding elevation above Lake Erie—

| | | | |
|---|---|---|---|
| Hillsborough, Highland Co., | 560. | Highland, west of Akron, | (same,) |
| West Union, Adams Co. | 410. | Huron Summit Swamp, | 414. |
| Yellow Springs, Green Co. (Cokely's door sill,) | 398. | Portage Summit, | 395. |
| Mahoning Summit Swamp (Champion, Trumbull County,) | 342. | Kilbuck Summit Swamp, (Harrisville, Medina County,) | 337. |
| St. Mary's Mercer County, (Canal Level,) | 278 | Olentangy, at Delaware, | 278. |
| | | Kilbuck, at mouth of Apple creek, near Wooster, | 278. |
| Scioto, at mouth of Mill Creek, | 271. | Newark, Old Court-House door sill, | 272. |

| | | | |
|---|---|---|---|
| Columbus, west door-sill, Capitol, | 197,5. | Walhonding, at mouth Kilbuck, | 197. |
| Sinking Spring, Adams County, | 150. | Putnam, Muskingum County, | 150. |
| Scioto, at Columbus Feeder, | 128,75. | Muskingum, at Dresden, | 127.61 |
| | | Ohio, at Beaver, Pa., | 127. |
| Fort Defiance, (Canal level) | 98 | Cleveland Court-House, | 95. |

It will be seen that the places compared are similarly situated, i. e. summits stand in contrast with summits, and streams and places upon them are collated with each other."

*Indiana.*—Dr. David Dale Owen is the Geologist of this young and rising state. We have before us his "Report of a Geological Reconnoisance," made in the year 1837, pp 34, which indicates, that Indiana is resolved not to lag behind her elder sister, in this important enterprize; and that she has confided it to gentlemen of science. We have room but for a single extract:

"None of the precious metals will ever be found in Indiana, unless in minute portions in boulders, or in small quantities in combination with other metals; because the primitive and grauwacke formations, in which alone productive mines of gold and silver ore occur, do not exist in Indiana. It is true, that in some rare instances, silver is found as a sulphuret and as red silver ore, in such formations as exist in the western country; but I have seen no symptoms of any such in our State. The same may be said of bismuth, tin ore, and native arsenic. The only metals which we need look for, are iron, lead, antimony, maganese, zinc, cobalt, and possibly some varieties of copper and arsenic ores.

It is not likely that anthracite coal will ever be found in Indiana; because that mineral is usually found in the primitive and grauwacke formations.

Several detached pieces of native copper have been found in the State, one weighing five pounds; but, from the nature of the ore, its occurring in washed gravels, and only in isolated pieces, I have reason to believe that they do not originate in the State. I may add that the kupferschiefer of the German miners, yields, at the mines of Mansfield, in Thuringia, an abundant supply of copper ore. This copper slate is found at the bottom of the new red sandstone formation, which overlies the bituminous coal formation; and copper ores have been found in the carboniferous and mountain limestone. There is therefore, a possibility of discovering workable copper ore in the formations of Indiana.

The fertility of the soil of Indiana is universally admitted, yet few are aware that it arises mainly from its geological position. It is well known to geologists, that that soil is the most productive, which has been derived from the destruction of the greatest variety of different rocks; for thus only is produced the due mixture of gravel, sand, clay, and limestone, necessary to form a good medium for the retension and transmission of the nutrative fluids, be they liquid or aeriform, to the roots of plants. Now, Indiana is situated near the middle of the Great Valley of Northwestern America, and far distant from the primitive range of mountains; and her soil is accordingly formed from the destruction of a vast variety of rocks, both crystalline and sedimentary, which have been minutely divided and intimately blended together by the action of air and water, It has all the elements, therefore, of extraordinary fertility."

*Michigan.*—The survey of this new state is only begun. The first report, from the pen of Dr. Douglas Houghton, the principal Geologist, is comprised in 37 pages; and was made to the Legislature of Michigan, on the 22d of January last. The departments of Botany and Zoology, are confided to Dr. Abraham Sager. Our limits do not permit us to extract any thing from this document; and, indeed, the time has not yet been sufficient to develope results. We cannot, however, dismiss the subject, without bearing the testimony of our humble admiration, to the enterprise of our young sister of the north, for having so soon after puberty, passed into gestation with such an important project.

We trust that geological surveys of all the Western and Southern states, will soon be undertaken. Tennessee set the example, and Dr. Troost has been for several years, engaged in the work. Kentucky, Illinois, Missouri, and the states bordering on the Gulph, require to be aroused to action; and their physicians might contribute more than any other class of the community, to the promotion of the object—an object in which the profession has a deep interest; as the medical history of a country, can never be properly written, before its geological constitution is known.

